# Datasets on demographic trends in enrollment into undergraduate engineering programs at Covenant University, Nigeria

**DOI:** 10.1016/j.dib.2018.02.073

**Published:** 2018-03-07

**Authors:** Segun I. Popoola, Aderemi A. Atayero, Joke A. Badejo, Jonathan A. Odukoya, David O. Omole, Priscilla Ajayi

**Affiliations:** a Department of Electrical and Information Engineering, Covenant University, Ota, Nigeria; b Department of Psychology, Covenant University, Ota, Nigeria; c Department of Civil Engineering, Covenant University, Ota, Nigeria; d Center for Systems and Information Services, Covenant University, Ota, Nigeria

**Keywords:** Analytics, Sustainable education, Higher education, Data mining, Engineering, Smart campus

## Abstract

In this data article, we present and analyze the demographic data of undergraduates admitted into engineering programs at Covenant University, Nigeria. The population distribution of 2649 candidates admitted into Chemical Engineering, Civil Engineering, Computer Engineering, Electrical and Electronics Engineering, Information and Communication Engineering, Mechanical Engineering, and Petroleum Engineering programs between 2002 and 2009 are analyzed by gender, age, and state of origin. The data provided in this data article were retrieved from the student bio-data submitted to the Department of Admissions and Student Records (DASR) and Center for Systems and Information Services (CSIS) by the candidates during the application process into the various engineering undergraduate programs. These vital information is made publicly available, after proper data anonymization, to facilitate empirical research in the emerging field of demographics analytics in higher education. A Microsoft Excel spreadsheet file is attached to this data article and the data is thoroughly described for easy reuse. Descriptive statistics and frequency distributions of the demographic data are presented in tables, plots, graphs, and charts. Unrestricted access to these demographic data will facilitate reliable and evidence-based research findings for sustainable education in developing countries.

**Specifications Table**

**Table:** 

Subject area	*Engineering Education*
More specific subject area	*Demographic Analytics*
Type of data	*Tables, charts, and spreadsheet file*
How data was acquired	*The demographic data were retrieved from the information submitted to the Department of Admissions and Student Records (DASR) and Center for Systems and Information Services (CSIS) by the candidates during the application process into the various engineering undergraduate programs.*
Data format	*Raw, analyzed*
Experimental factors	*Applicants with incomplete academic records were excluded*
Experimental features	*Descriptive statistics and frequency distributions of the demographic data are analyzed and presented in tables and charts.*
Data source location	*Covenant University, Canaanland, Ota, Nigeria (Latitude 6.6718° N, Longitude 3.1581° E)*
Data accessibility	*In order to encourage evidence-based research in admission analytics, detailed datasets are made publicly available in a Microsoft Excel spreadsheet file attached to this article.*

**Value of the data**

• Demographic data provided in this article will encourage empirical research and the adoption of data analytics in understanding the trends in enrollment of undergraduates in higher education, especially in developing countries [1], [2], [3], [4], [5].
• Unrestricted access to these demographic data will give executives, management, and policy makers in higher education useful insights for better decision-making [6], [7].
• Further exploration of these data by the global educational research community will facilitate gender equality in higher education and encourage women participation in the field of engineering. Also, underserved population can be identified and possible solutions may be recommended to relevant authorities [8], [9], [10], [11], [12], [13].
• Descriptive statistics and frequency distributions that are presented in tables and charts will make data interpretation much easier for scientific conclusions [14], [15], [16], [17].
• Data shared in this data article will open up doors for new research collaborations.

## 1 Data

The fourth goal (Goal 4) of the Sustainable Development Goals (SDGs) set by the general assembly of the United Nations in September 2015 focus on “ensuring inclusive and equitable quality education, and promoting lifelong learning opportunities for all” [18], [19], [20]. It is expected that both women and men should have equal access to “affordable and quality technical, vocational, tertiary education” by 2030. This, in essence, will encourage gender equality in higher education, most especially for men-dominated programs of study.

**Fig. 1:**
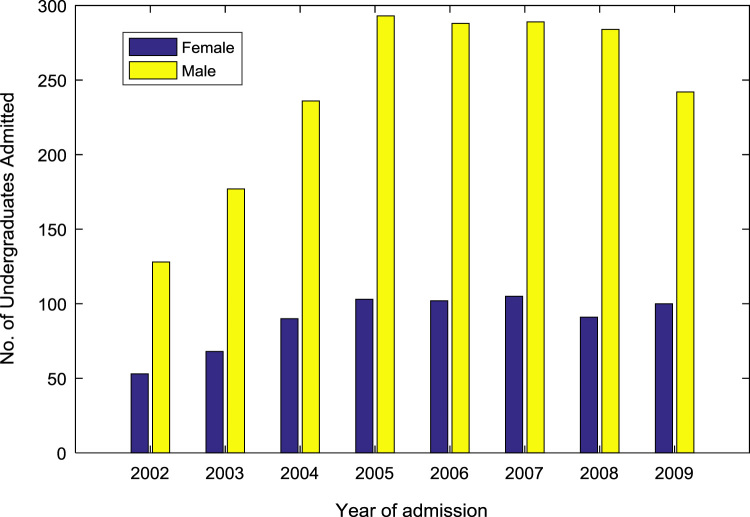
Bar chart showing the gender distribution of undergraduates admitted into engineering programs.

**Fig. 2:**
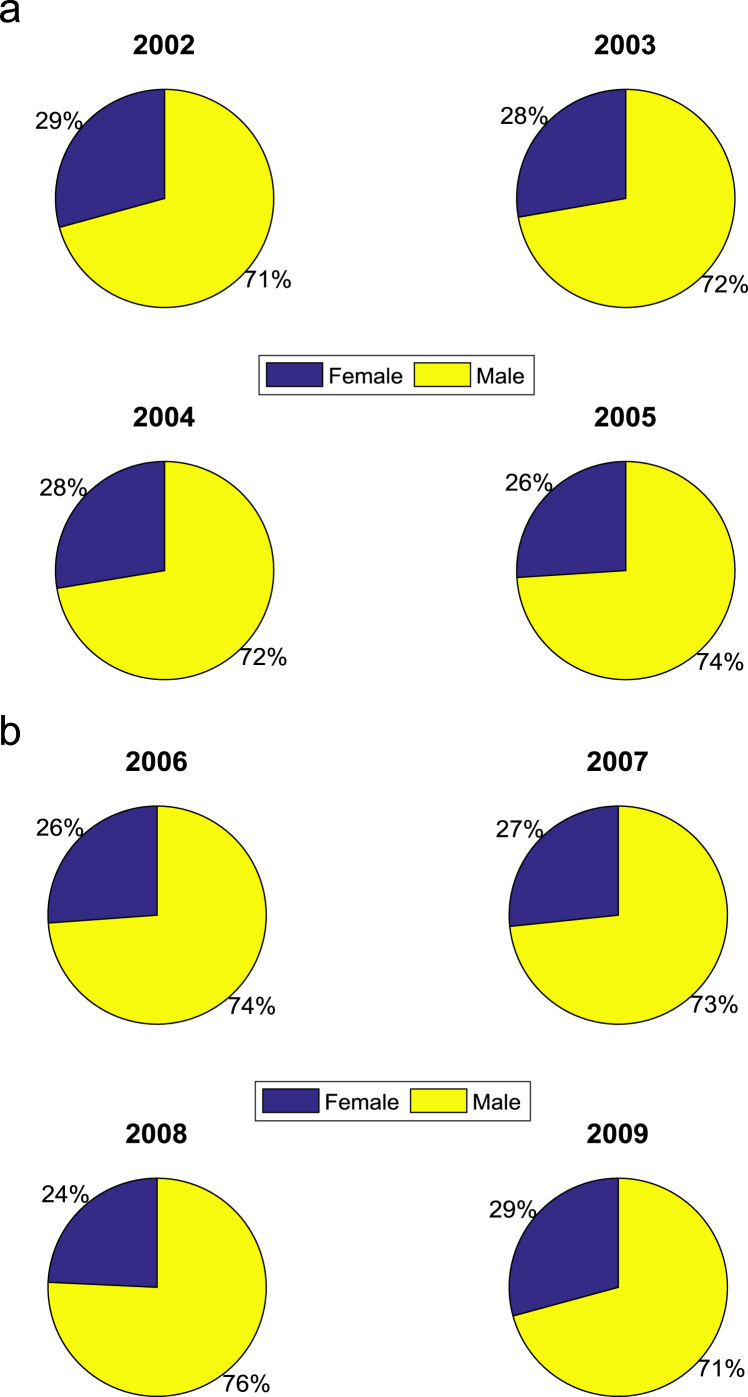
(a)–(b). Proportions of female and male undergraduates admitted (2002–2009).

Table 1 presents the gender distribution of undergraduates admitted into the seven engineering programs (Chemical Engineering, Civil Engineering, Computer Engineering, Electrical and Electronics Engineering, Information and Communication Engineering, Mechanical Engineering, and Petroleum Engineering) over the period of eight years (2002–2009). In each year, the engineering programs are significantly male-dominated. Fig. 1 shows a good graphical visualization of the gender distribution of undergraduates admitted into the seven engineering programs in the eight-year period. The proportions of female to male undergraduates in the engineering programs are illustrated in Fig. 2(a)–(b).

**Table 1:** Gender distribution of undergraduates admitted into engineering programs.

Year of admission	No. of female students	No. of male students	Total no. of students
2002	53	128	181
2003	68	177	245
2004	90	236	326
2005	103	293	396
2006	102	288	390
2007	105	289	394
2008	91	284	375
2009	100	242	342
*Total*	712	1937	2649

**Fig. 3:**
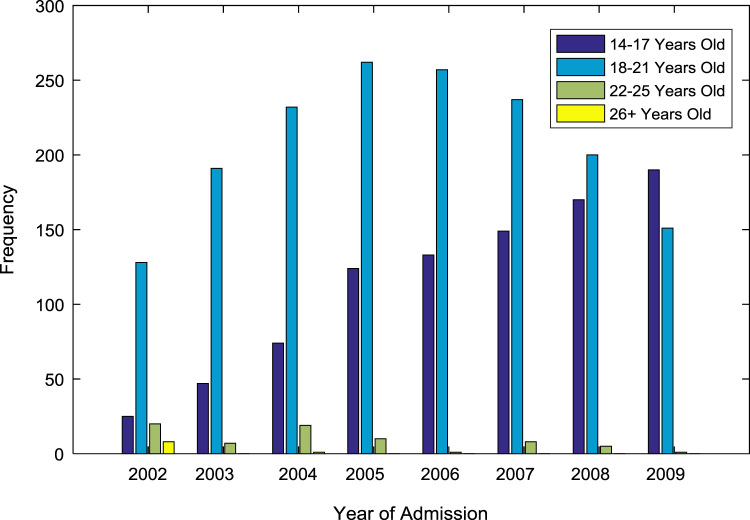
Bar chart showing the age distribution of undergraduates admitted into engineering programs.

**Fig. 4:**
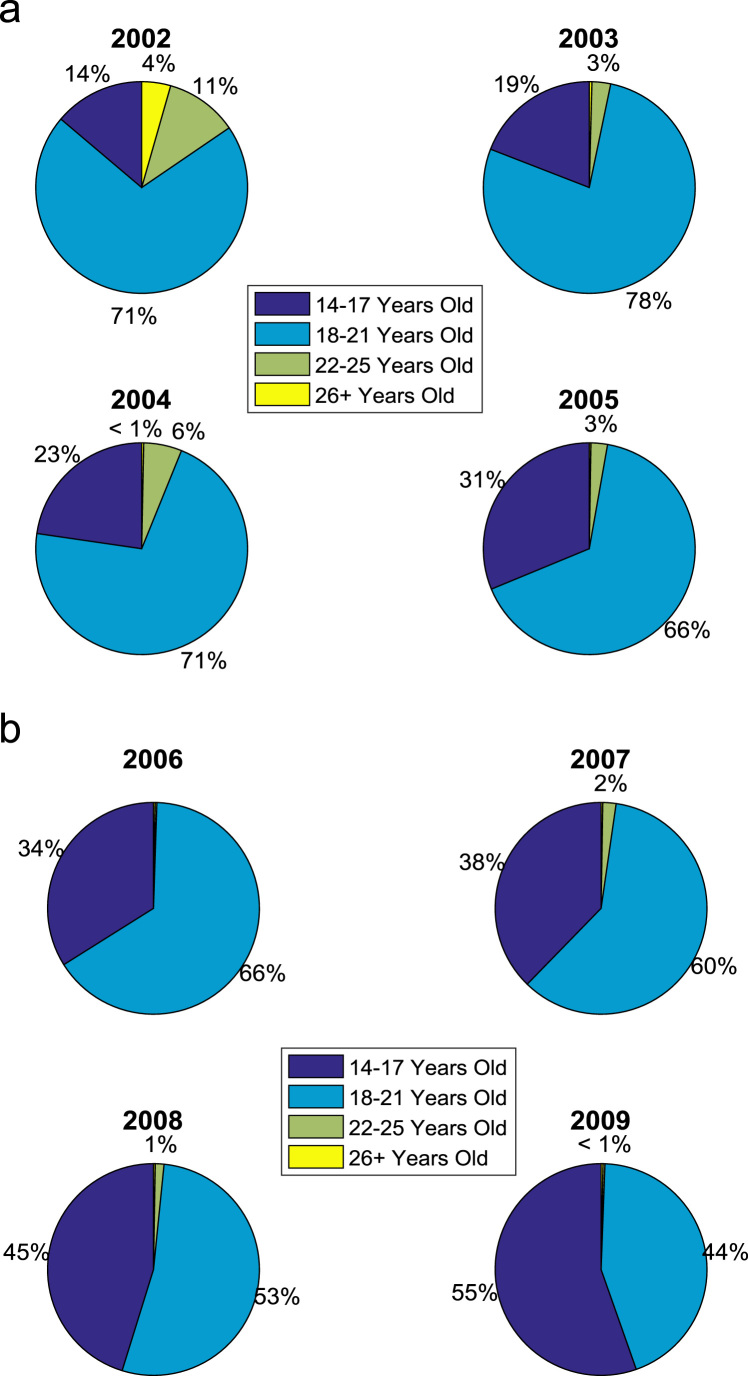
(a)–(b). Proportions of undergraduates admitted by age (2002–2009).

In addition, age distribution of the undergraduates admitted into the engineering programs at Covenant University are presented and analyzed. The ages of the students are grouped into four categories: 14–17 years old; 18–21 years old; 22–25 years old; and 26 years old and above. The population distribution of the undergraduates by age is presented in Table 2. The bar chart in Fig. 3 shows the graphical visualization of the age distribution. The proportions of undergraduates each of the age groups are shown in Fig. 4(a)–(b).

**Table 2:** Age distribution of undergraduates admitted into engineering programs.

Year of admission	Entry age in years			
	*14–17*	*18–21*	*22–25*	*26 and above*
2002	25	128	20	8
2003	47	191	7	0
2004	74	232	19	1
2005	124	262	10	0
2006	133	257	0	0
2007	149	237	8	0
2008	170	200	5	0
2009	190	151	1	0
*Total*	912	1658	70	9

## 2 Experimental design, materials and methods

For the eight-year admission period covered in this study, the demographic data (gender, age, and state of origin) of undergraduate admitted into the seven engineering programs available at Covenant University, Nigeria were retrieved from the student bio-data submitted to the Department of Admissions and Student Records (DASR) and Center for Systems and Information Services (CSIS). The population distribution of 2649 candidates admitted into Chemical Engineering, Civil Engineering, Computer Engineering, Electrical and Electronics Engineering, Information and Communication Engineering, Mechanical Engineering, and Petroleum Engineering programs between 2002 and 2009 are analyzed by gender, age, and state of origin. Descriptive statistics and frequency distributions that are presented in tables and graphs will make data interpretation much easier for scientific conclusions.

**Fig. 5:**
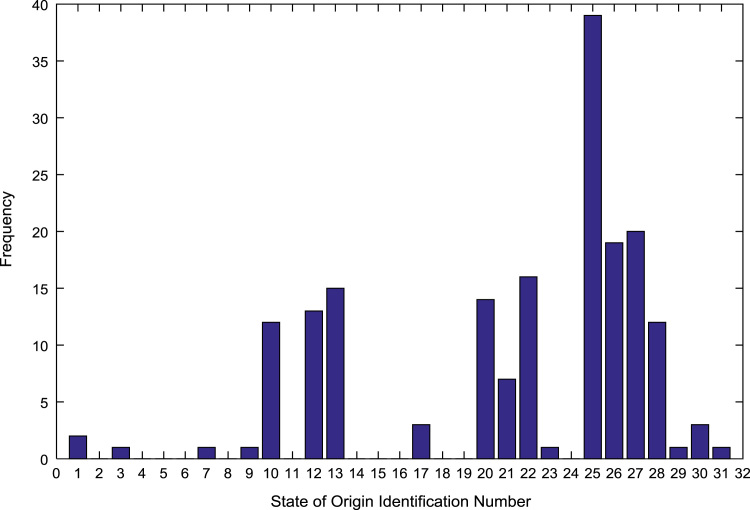
Frequency distribution by state of origin for year 2002.

**Fig. 6:**
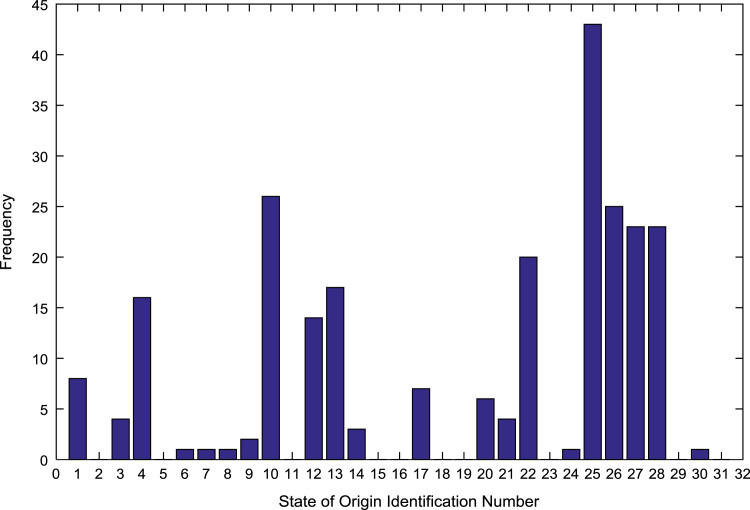
Frequency distribution by state of origin for year 2003.

**Fig. 7:**
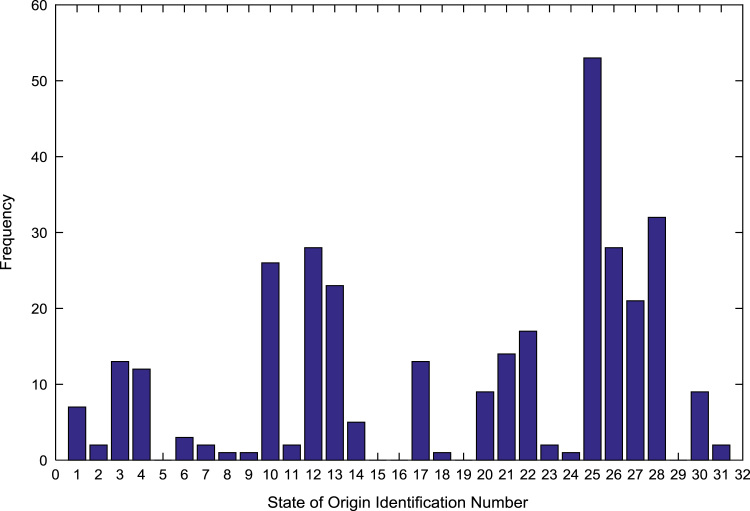
Frequency distribution by state of origin for year 2004.

**Fig. 8:**
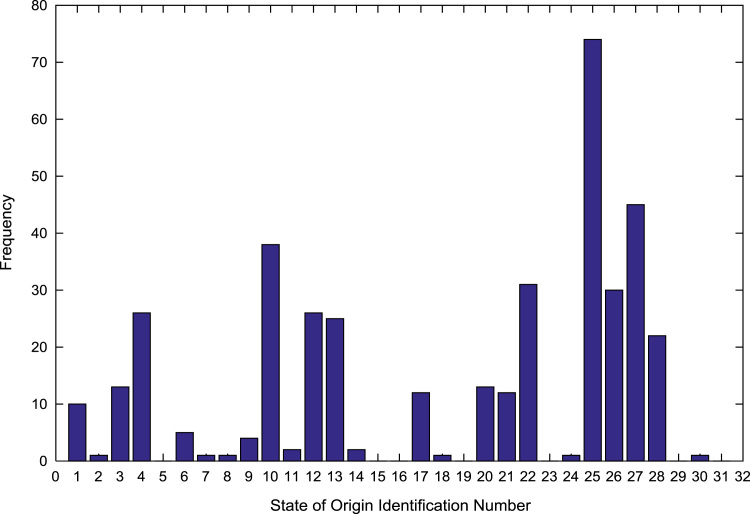
Frequency distribution by state of origin for year 2005.

**Fig. 9:**
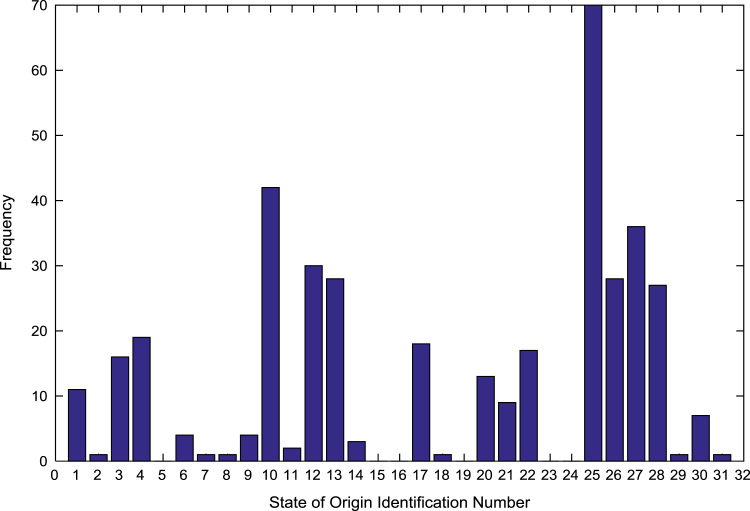
Frequency distribution by state of origin for year 2006.

**Fig. 10:**
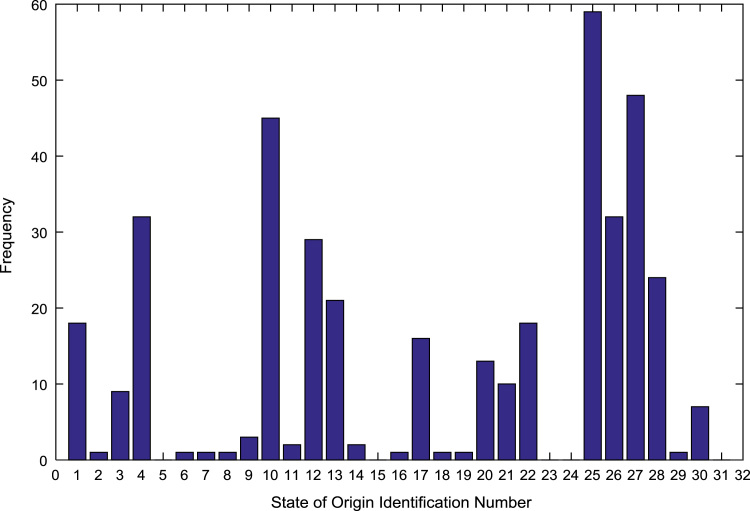
Frequency distribution by state of origin for year 2007.

**Fig. 11:**
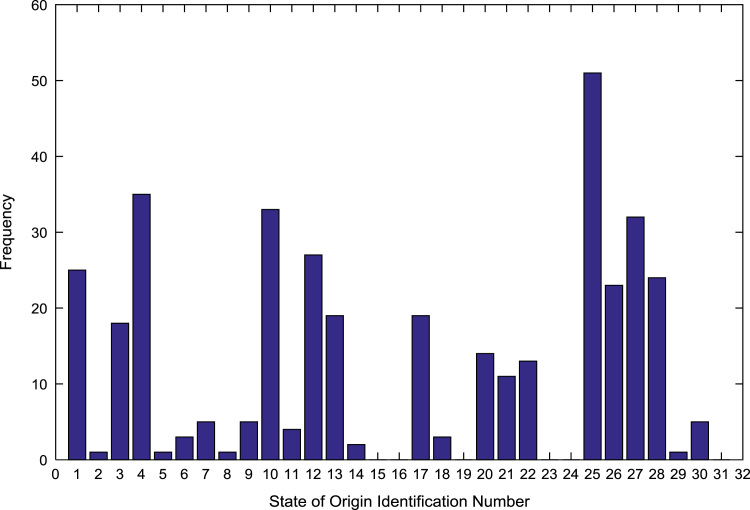
Frequency distribution by state of origin for year 2008.

**Fig. 12:**
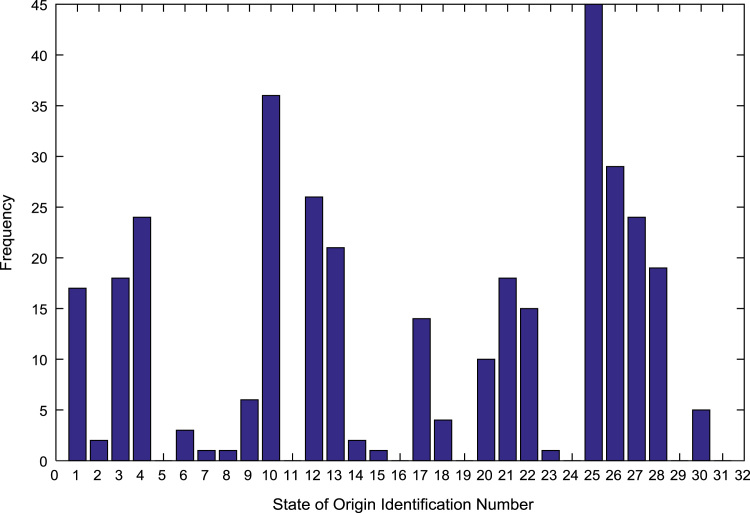
Frequency distribution by state of origin for year 2009.

The population sample of the undergraduates admitted into the engineering programs are analyzed by state of origin and the results are presented in Table 3. All of the states of the Federation and the Federal Capital Territory (FCT) are represented except Jigawa, Katsina, Kebbi, Sokoto, Yobe, and Zamfara states. Fig. 5, Fig. 6, Fig. 7, Fig. 8, Fig. 9, Fig. 10, Fig. 11, Fig. 12 illustrate the frequency distributions of the undergraduates in engineering programs by state of origin from 2002 to 2009 respectively.

**Table 3:** Distribution of undergraduates admitted into engineering programs by state of origin.

State ID	State of origin	2002	2003	2004	2005	2006	2007	2008	2009	Total	%
1	Abia	2	8	7	10	11	18	25	17	98	3.69
2	Adamawa	0	0	2	1	1	1	1	2	8	0.30
3	Akwa-Ibom	1	4	13	13	16	9	18	18	92	3.47
4	Anambra	0	16	12	26	19	32	35	24	164	6.18
5	Bauchi	0	0	0	0	0	0	1	0	1	0.04
6	Bayelsa	0	1	3	5	4	1	3	3	20	0.75
7	Benue	1	1	2	1	1	1	5	1	13	0.49
8	Borno	0	1	1	1	1	1	1	1	7	0.26
9	Cross River	1	2	1	4	4	3	5	6	26	0.98
10	Delta	12	26	26	38	42	45	33	36	258	9.72
11	Ebonyi	0	0	2	2	2	2	4	0	12	0.45
12	Edo	13	14	28	26	30	29	27	26	193	7.27
13	Ekiti	15	17	23	25	28	21	19	21	169	6.37
14	Enugu	0	3	5	2	3	2	2	2	19	0.72
15	FCT	0	0	0	0	0	0	0	1	1	0.04
16	Gombe	0	0	0	0	0	1	0	0	1	0.04
17	Imo	3	7	13	12	18	16	19	14	102	3.84
18	Kaduna	0	0	1	1	1	1	3	4	11	0.41
19	Kano	0	0	0	0	0	1	0	0	1	0.04
20	Kogi	14	6	9	13	13	13	14	10	92	3.47
21	Kwara	7	4	14	12	9	10	11	18	85	3.20
22	Lagos	16	20	17	31	17	18	13	15	147	5.54
23	Nasarawa	1	0	2	0	0	0	0	1	4	0.15
24	Niger	0	1	1	1	0	0	0	0	3	0.11
25	Ogun	39	43	53	74	70	59	51	45	434	16.36
26	Ondo	19	25	28	30	28	32	23	29	214	8.07
27	Osun	20	23	21	45	36	48	32	24	249	9.39
28	Oyo	12	23	32	22	27	24	24	19	183	6.90
29	Plateau	1	0	0	0	1	1	1	0	4	0.15
30	Rivers	3	1	9	1	7	7	5	5	38	1.43
31	Taraba	1	0	2	0	1	0	0	0	4	0.15

**Fig. 13:**
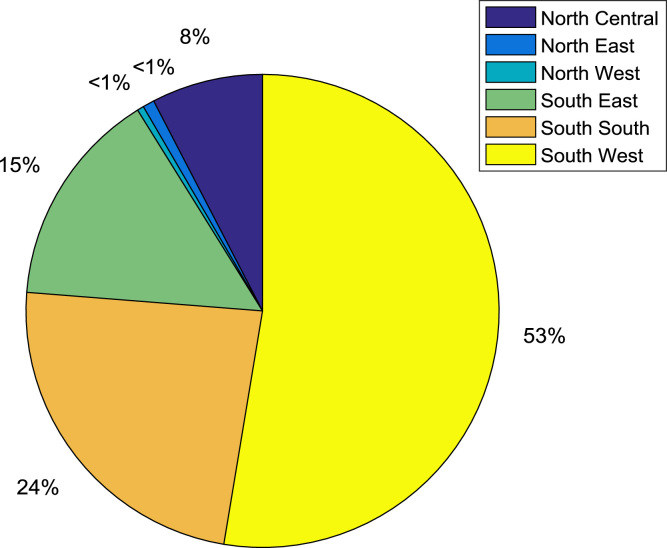
Percentage of undergraduates in engineering programs by geopolitical zones.

Economic, political, and educational resources are often shared across six geopolitical zones in Nigeria. The states of the federation are grouped into the six geopolitical zones as presented in Table 4. The analysis of the contributions of each zone to the total number of engineering undergraduates are also available in Table 4. Fig. 13 shows the percentage contribution of each zone to the total number of undergraduates admitted into engineering programs at Covenant University, Nigeria.

**Table 4:** Distribution of undergraduates in engineering programs across the six geopolitical zones.

Geopolitical zones	States of the federation	Total no. of undergraduates in engineering programs	Total no. of undergraduates in geopolitical zone
North Central	Benue	13	202
	Kogi	92	
	Kwara	85	
	Nasarawa	4	
	Niger	3	
	Plateau	4	
	FCT	1	
			
North East	Adamawa	8	21
	Bauchi	1	
	Borno	7	
	Gombe	1	
	Taraba	4	
	Yobe	0	
			
North West	Jigawa	0	12
	Kaduna	11	
	Kano	1	
	Katsina	0	
	Kebbi	0	
	Sokoto	0	
	Zamfara	0	
			
South East	Abia	98	395
	Anambra	164	
	Ebonyi	12	
	Enugu	19	
	Imo	102	
			
South South	Akwa-Ibom	92	627
	Cross River	26	
	Bayelsa	20	
	Rivers	38	
	Delta	258	
	Edo	193	
			
South West	Ekiti	169	1396
	Lagos	147	
	Ogun	434	
	Ondo	214	
	Osun	249	
	Oyo	183	

## 3 Conclusion

This data article presented and analyzed the demographic trends in enrollment into undergraduate engineering programs at Covenant University, Nigeria. Demographic data provided in this article will encourage empirical research and the adoption of data analytics in understanding the trends in enrollment of undergraduates in higher education, especially in developing countries. Descriptive statistical analyses were performed based on gender, age, and state of origin of the population sample. Evidence-based insights gained from these data will inform proper formulation of admission policies that govern entry into engineering programs in the sub-Saharan African region. The contribution of these data is considered to be significant in the sense that it revealed the need to advocate for the recruitment and retention of women in technical disciplines in developing countries. Free accessibility to these demographic data will give executives, management, and policy makers in higher education useful insights for better decision-making.
